# Neoadjuvant PD-1/VEGF bispecific antibody combined with chemotherapy for locally advanced pulmonary lymphoepithelioma-like carcinoma achieving pathological complete response: a case report and literature review

**DOI:** 10.3389/fimmu.2026.1826272

**Published:** 2026-06-12

**Authors:** Yuanli You, Mingxing Yang, Zhiwei Chen, Xiayu Wu, Wen Dong

**Affiliations:** 1 Department of Respiratory Medicine, Hainan Cancer Hospital, Haikou, China; 2 Department of Pathology, Hainan Cancer Hospital, Haikou, China

**Keywords:** case report, ivonescimab, neoadjuvant therapy, PD-1/VEGF bispecific antibody, primary pulmonary lymphoepithelioma-like carcinoma

## Abstract

Primary pulmonary lymphoepithelioma-like carcinoma (PPLELC) is a rare Epstein-Barr virus (EBV)-associated subtype of non-small cell lung cancer (NSCLC), accounting for less than 1% of all NSCLC cases. Because prospective data are scarce, no standardized perioperative strategy has been established. We report the case of a 68-year-old female never-smoker diagnosed with stage IIIC PPLELC (cT3N3M0). The patient received four cycles of neoadjuvant therapy consisting of gemcitabine plus cisplatin in combination with ivonescimab, a novel PD-1/VEGF bispecific antibody, followed by radical surgical resection. Postoperative pathological examination revealed no residual viable tumor cells, indicating a pathological complete response (pCR). At the latest follow-up (March 2026, approximately eight months after diagnosis), no radiological evidence of recurrence or distant metastasis was observed. To our knowledge, this is the first reported case of neoadjuvant PD-1/VEGF bispecific antibody combined with chemotherapy in locally advanced PPLELC, that achieved both pCR and R0 resection. This case suggests the potential feasibility and antitumor activity of neoadjuvant ivonescimab combined with chemotherapy in locally advanced PPLELC.

## 1 Introduction

Pulmonary lymphoepithelioma-like carcinoma (PPLELC) is a rare subtype of primary lung cancer that is strongly associated with Epstein–Barr virus (EBV) infection and predominantly occurs in Asian, younger, and never-smoking populations (1). Owing to its extremely low incidence, prospective large-scale clinical trials are lacking, and therapeutic strategies are largely extrapolated from treatment paradigms established for non-small cell lung cancer (NSCLC) (2).

For early-stage disease, complete surgical resection remains the cornerstone of curative treatment. In contrast, management of locally advanced (stage III) disease aims to reduce recurrence risk through multimodal strategies incorporating systemic therapy and radical resection when feasible. Platinum-based doublet chemotherapy historically served as an important systemic treatment platform for locally advanced NSCLC. In the current immunotherapy era, however, chemoimmunotherapy and other immunotherapy-based regimens have increasingly become central components of multimodal treatment strategies.

In recent years, immune checkpoint inhibitors (ICIs) have fundamentally reshaped the therapeutic landscape of advanced NSCLC. Given its distinctive tumor immune microenvironment—characterized by dense lymphocytic infiltration and a strong association with EBV—PPLELC is hypothesized to exhibit increased immunogenicity and enhanced responsiveness to immunotherapy. Nevertheless, clinical evidence supporting perioperative immunotherapy in PPLELC remains exceedingly limited.

Ivonescimab is a tetravalent, Fc-silent bispecific antibody designed to block programmed cell death protein 1 (PD-1) and vascular endothelial growth factor A (VEGF-A) simultaneously. Its dual-target design may combine restoration of exhausted T-cell activity with reversal of VEGF-mediated immunosuppression, including abnormal tumor vasculature, impaired dendritic-cell maturation, and limited effector-cell trafficking (3).

Herein, we report a case of locally advanced (stage IIIC) EBV-positive PPLELC that achieved marked radiological regression and ultimately pathological complete response (pCR) following neoadjuvant therapy with ivonescimab combined with platinum-based chemotherapy. This case illustrates the potential feasibility, pathological response, and mechanistic rationale of dual PD-1/VEGF blockade in the perioperative management of a rare lung cancer subtype.

## 2 Case presentation

### 2.1 Initial presentation and diagnostic workup

A 68-year-old female never-smoker presented on July 16, 2025, with cough, expectoration, and hemoptysis. She had no significant past medical history and no family history of malignancy.

Chest computed tomography (CT) revealed a well-defined soft-tissue mass in the right lower lobe measuring approximately 5.2 × 3.8 cm at its largest cross-sectional dimension. The lesion was associated with bronchial obstruction and adjacent interlobar pleural thickening. Multiple enlarged lymph nodes were identified in the mediastinum and right hilum, with the largest measuring approximately 1.1 cm in short-axis diameter, raising strong suspicion for primary lung malignancy. Baseline staging CT showed a right lower lobe mass with obstructive pneumonia, which had increased compared with previous imaging. Multiple enlarged lymph nodes were observed in the mediastinum and right hilum. Ultrasonography of the cervical and supraclavicular regions revealed abnormal lymph node structures in the bilateral supraclavicular areas, raising suspicion of metastatic involvement.

**Figure 1:**
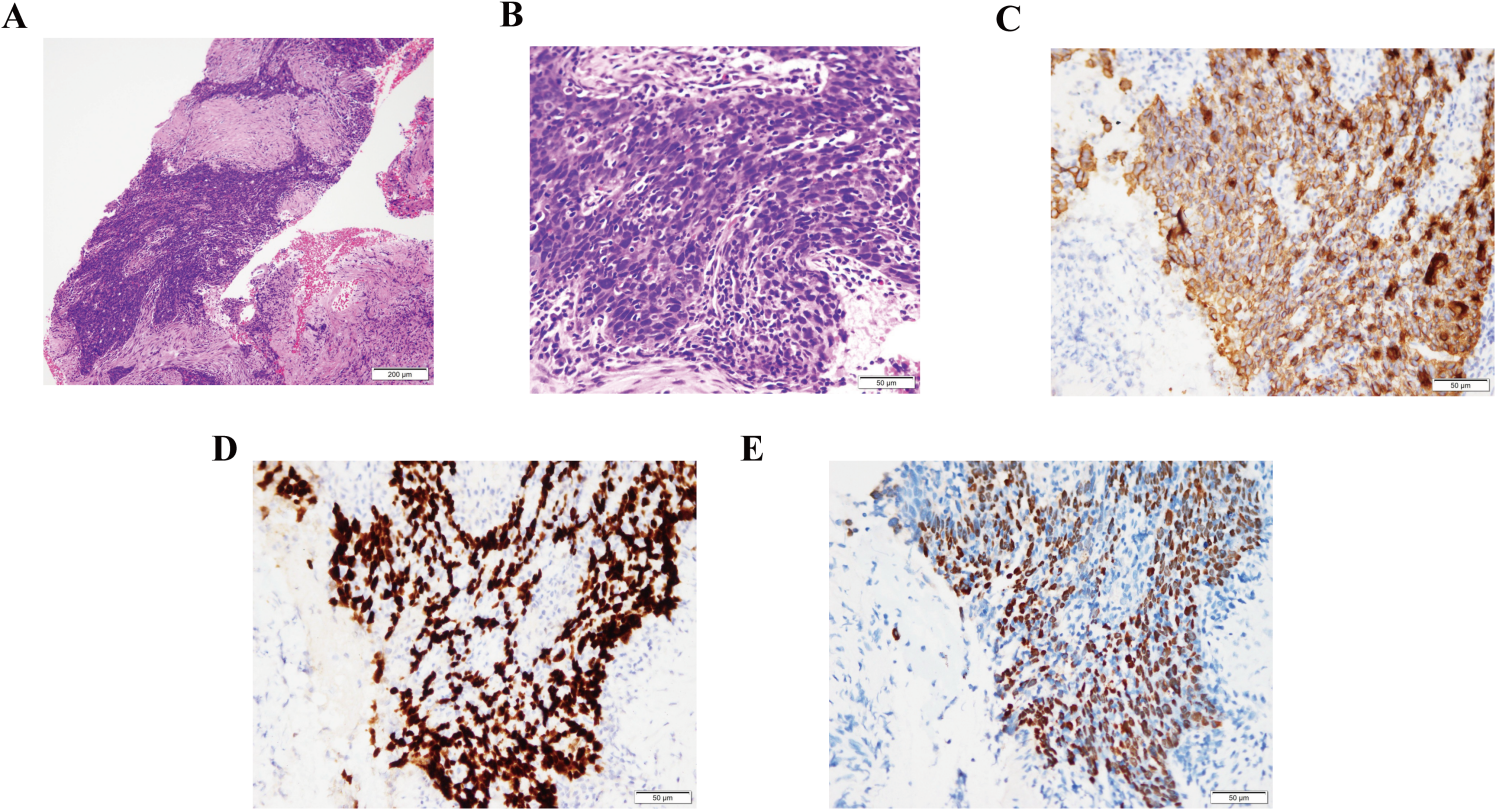
Preoperative biopsy histopathology and immunohistochemical staining. **(A)** Malignant tumor cells arranged in nests and sheets within the biopsy specimen (hematoxylin and eosin [H&E], ×100). **(B)** Tumor cells exhibiting spindle-shaped morphology, accompanied by prominent lymphocytic infiltration and reactive stromal fibrosis (H&E, ×400). **(C)** Immunohistochemistry showing cytoplasmic positivity for CK5/6 (×400). **(D)** Immunohistochemistry demonstrating nuclear positivity for p63 (×400). **(E)** *In situ* hybridization revealing nuclear positivity for EBV-encoded small RNA (EBER) (×400).

CT-guided percutaneous lung biopsy was subsequently performed. Histopathological examination demonstrated carcinoma with prominent lymphoid stroma, consistent with lymphoepithelioma-like carcinoma. Immunohistochemistry (IHC) results were as follows: CK7 (−), TTF-1 (−), Napsin A (−), p40 (−), p63 (+), CK5/6 (+), Ki-67 (approximately 30%+), c-Met (5% strong+, 20% weak+), ALK (PS0) (−), CD56 (−), synaptophysin (−), chromogranin A (−), and CD21 (−). *In situ* hybridization confirmed positivity for EBV-encoded small RNA (EBER). These findings supported the diagnosis of pulmonary lymphoepithelioma-like carcinoma (PPLELC) (**Figures 1A–E**). The main differential diagnoses included lung squamous cell carcinoma, metastatic nasopharyngeal carcinoma, small-cell lung cancer, and pulmonary metastatic lymphoepithelioma-like carcinoma from extrapulmonary sites. Lung adenocarcinoma was considered unlikely because TTF-1 and Napsin A were negative. Neuroendocrine carcinoma was not supported by the absence of CD56, synaptophysin, and chromogranin A expression. Metastatic nasopharyngeal carcinoma was excluded based on the absence of nasopharyngeal lesions on clinical and imaging evaluation. The combination of lymphoepithelioma-like morphology, CK5/6 and p63 positivity, EBER positivity, and the absence of another primary site supported the diagnosis of primary pulmonary lymphoepithelioma-like carcinoma.

Comprehensive staging evaluation revealed no evidence of distant metastasis. Based on the right lower lobe primary lesion, mediastinal and right hilar lymphadenopathy, and suspected bilateral supraclavicular lymph node involvement, the patient was clinically staged as stage IIIC (cT3N3M0). Serum lung cancer–related tumor markers were within normal limits. Molecular profiling was performed using next-generation sequencing (NGS), which identified no actionable driver alterations, including EGFR, ALK, ROS1, RET, MET, KRAS, or BRAF alterations. Programmed death-ligand 1 (PD-L1) expression, assessed using the 22C3 assay, demonstrated a tumor proportion score (TPS) of 70%.

### 2.2 Multidisciplinary evaluation and neoadjuvant strategy

Following multidisciplinary team (MDT) discussion, upfront surgery was deemed technically challenging with a low likelihood of achieving R0 resection due to extensive nodal involvement (N3 disease). Given the high PD-L1 expression (TPS 70%) and the immunogenic features of EBV-associated PPLELC, neoadjuvant immunotherapy combined with chemotherapy was recommended to facilitate tumor downstaging, increase the probability of complete resection, and eradicate potential micrometastatic disease.

From August 6 to October 20, 2025, the patient received four 21-day cycles of neoadjuvant gemcitabine plus cisplatin combined with ivonescimab. Each cycle consisted of gemcitabine 1,000 mg/m² intravenously on days 1 and 8, cisplatin 75 mg/m² intravenously on day 1, and ivonescimab 20 mg/kg intravenously on day 1. All planned cycles were completed without dose reduction, treatment delay, or discontinuation. During neoadjuvant therapy, the patient remained clinically stable, with good treatment adherence and no clinically significant adverse events requiring intervention. According to CTCAE criteria, no grade 3 or higher treatment-related adverse events and no definite immune-related adverse events were observed. The selection of this regimen was based on multiple clinical and biological considerations. Gemcitabine and cisplatin constitute a well-established first-line chemotherapy backbone for advanced non-small cell lung cancer and are also widely used in nasopharyngeal carcinoma, a malignancy that shares histopathological characteristics and EBV association with PPLELC. Given these similarities, this platinum-based doublet was considered a rational cytotoxic platform. Ivonescimab, a bispecific antibody targeting both PD-1 and VEGF, was incorporated to enhance antitumor efficacy through concurrent immune checkpoint blockade and anti-angiogenic modulation. Inhibition of VEGF signaling has been shown to normalize tumor vasculature, improve immune cell trafficking, and alleviate immunosuppressive signaling within the tumor microenvironment, thereby potentially augmenting PD-1–mediated immune activation. Furthermore, accumulating evidence from locally advanced NSCLC indicates that neoadjuvant chemoimmunotherapy based on PD-1 inhibitors can achieve high pathological response rates and improve surgical outcomes, providing additional support for adopting this strategy in the present case.

### 2.3 Surgical management and pathological assessment

**Figure 2:**
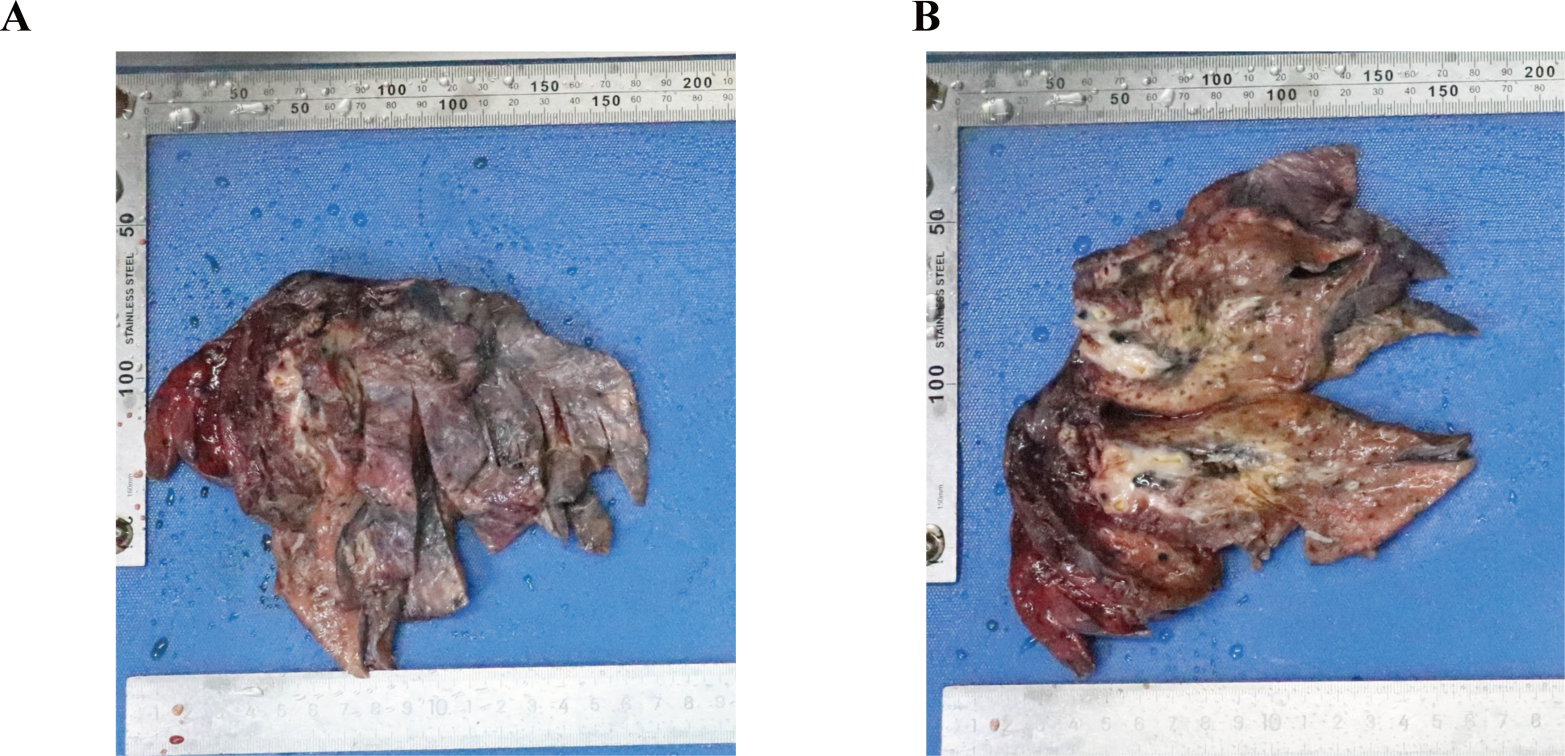
Gross appearance of the surgically resected specimen. The tumor bed area was grayish-yellow and firm in texture, measuring 4.5 × 3.5 × 3.3 cm, with ill-defined margins and focal cystic cavity formation **(A, B)**.

After four cycles of neoadjuvant therapy, preoperative PET-CT demonstrated regression of the right lower lobe lesion with mild residual radiotracer uptake, suggesting limited residual tumor activity. Multiple mediastinal and right hilar lymph nodes still showed increased uptake, whereas no extra-thoracic metastasis was detected. Based on radiological tumor regression, absence of distant metastasis, and overall surgical operability, radical surgical resection was considered feasible after multidisciplinary reassessment. On November 20, 2025, the patient underwent video-assisted thoracoscopic right lower lobectomy with mediastinal lymph node dissection, pleural adhesion lysis, and intercostal nerve block. The resected specimen measured 14.6 × 12.3 × 4.8 cm (**Figures 2A, B**).

Postoperative pathological examination revealed fibrous tissue proliferation, histiocytic and multinucleated giant cell infiltration, extensive inflammatory cell infiltration, focal necrosis, calcification, and cholesterol clefts within the lung parenchyma. Atypical hyperplasia of adjacent alveolar epithelium and marked interstitial fibrosis were observed. Importantly, no viable tumor cells were identified in the primary lesion or in regional lymph nodes. The neoadjuvant immunotherapy-related response assessment showed severe inflammatory infiltration, tertiary lymphoid structure formation, and neovascularization within the tumor bed.

**Figure 3:**
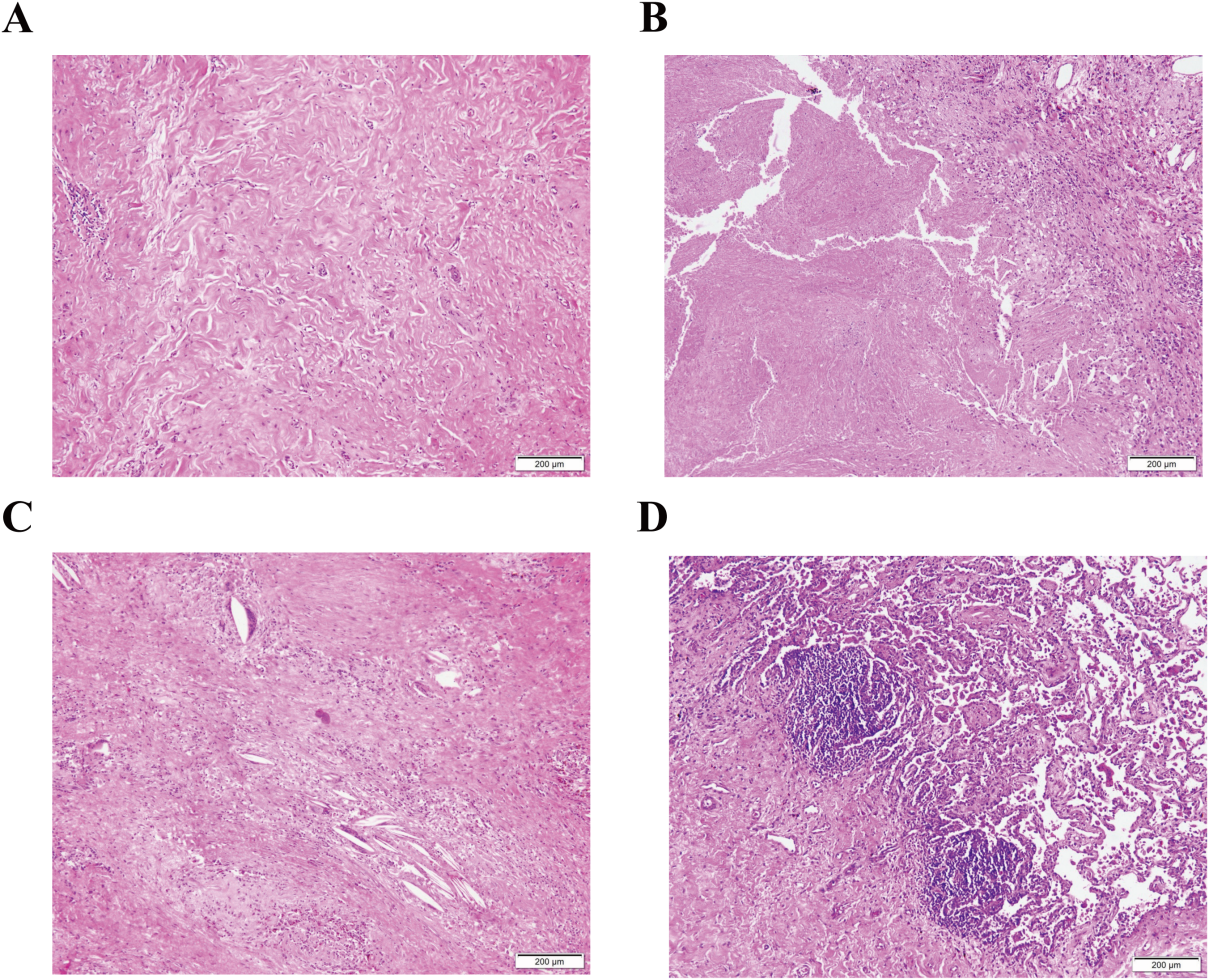
Histopathological findings of the radical resection specimen after neoadjuvant therapy. **(A)** Fibrotic area within the tumor bed. **(B)** Necrotic region identified in the tumor bed. **(C)** Prominent inflammatory cell infiltration with focal cholesterol cleft formation in the tumor bed. **(D)** Tertiary lymphoid structure formation observed following neoadjuvant immunotherapy.

These findings were consistent with post-treatment changes and confirmed pathological complete response (pCR) with negative surgical margins (R0 resection). IHC demonstrated CD68 (focal+), CK5/6 (−), TTF-1 (positive in alveolar epithelium), and Ki-67 (approximately 1%+) (**Figures 3A–D**).

### 2.4 Follow-up and ongoing management

The postoperative course was uneventful. At the latest follow-up on March 17, 2026, chest CT demonstrated complete absorption of the postoperative cavity, with no evidence of local recurrence or distant metastasis.

**Figure 4:**
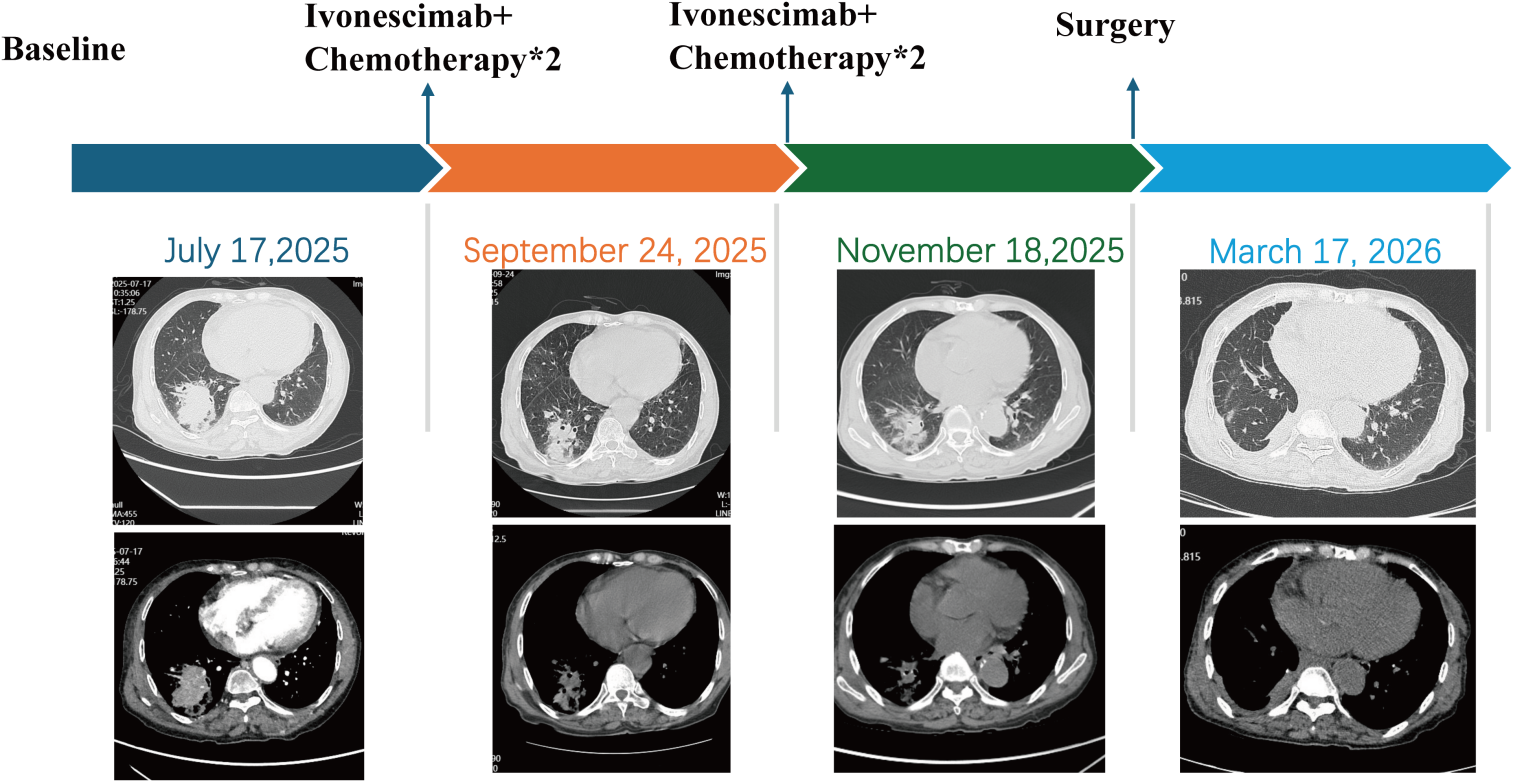
Timeline of treatment course and radiological evaluations from July 2025 to March 2026. CT, computed tomography.

Based on MDT recommendations and emerging clinical evidence supporting sustained immunotherapy in high-risk locally advanced disease, adjuvant ivonescimab therapy is planned to mitigate long-term relapse risk. A schematic overview of the treatment timeline and radiological response is presented in **Figure 4**.

## 3 Discussion

Primary pulmonary lymphoepithelioma-like carcinoma (PPLELC) is a distinct malignant epithelial neoplasm of the lung with a strong etiological association with Epstein–Barr virus (EBV) infection, particularly among Asian never-smokers. Early-stage disease is frequently asymptomatic, and imaging findings lack specific radiographic hallmarks, often leading to delayed diagnosis (4, 5). Although PPLELC is generally associated with more favorable overall survival than other subtypes of non-small cell lung cancer (NSCLC), outcomes remain unsatisfactory in advanced disease. Notably, patients with stage IV PPLELC exhibit a reported 5-year survival rate of approximately 9.6%, comparable to metastatic NSCLC (6, 7). These observations underscore the urgent need to optimize therapeutic strategies for locally advanced and metastatic disease.

Histopathological examination remains the diagnostic gold standard. Microscopically, PPLELC is characterized by undifferentiated carcinoma cells accompanied by dense infiltration of lymphocytes, plasma cells, and other inflammatory cells within the tumor stroma, forming a prototypical “immune-inflammatory” tumor microenvironment (TME) (8). This microenvironment is typically enriched with tumor-infiltrating lymphocytes (TILs), suggesting an intrinsically immunogenic tumor phenotype. Immunohistochemically, tumor cells frequently express squamous-associated cytokeratins such as CK5/6 and p63, whereas TTF-1 and Napsin A are commonly negative. Detection of EBV-encoded small RNA (EBER) by *in situ* hybridization represents a pivotal diagnostic criterion and confirms latent EBV infection within tumor cells (9). EBV-driven oncogenesis is believed to contribute to immune modulation, genomic instability, and tumor progression. Interestingly, EBER-positive tumors tend to present at a relatively later age and have been associated with improved prognosis compared with EBER-negative counterparts (4), further emphasizing the biological heterogeneity of this entity.

Emerging evidence indicates that EBV-positive PPLELC frequently exhibits elevated programmed death-ligand 1 (PD-L1) expression, with reported positivity rates ranging from 63.3% to 75.8% (10). Mechanistically, EBV infection may upregulate PD-L1 expression through inflammatory signaling pathways and viral oncoprotein–mediated immune modulation, thereby facilitating immune escape (4, 11). High PD-L1 expression is often accompanied by substantial peritumoral T-cell infiltration, reflecting an adaptive immune resistance phenotype. The coexistence of EBV positivity and high PD-L1 expression may therefore create a tumor microenvironment particularly amenable to immune checkpoint inhibition. In the present case, the tumor demonstrated a PD-L1 tumor proportion score (TPS) of 70%, providing a biologically plausible rationale for immune-based therapeutic intervention.

Surgical resection remains the cornerstone of treatment for early-stage PPLELC, and retrospective analyses have demonstrated favorable disease-free and overall survival among patients undergoing radical surgery. However, due to the rarity of the disease and the absence of prospective trials, there is no standardized therapeutic framework for locally advanced or metastatic PPLELC. Multidisciplinary treatment approaches incorporating chemotherapy, radiotherapy, and immunotherapy are generally adopted on the basis of extrapolation from NSCLC management principles.

PPLELC shares biological and pathological similarities with nasopharyngeal carcinoma and is therefore considered relatively radiosensitive (12, 13). The tumor also appears responsive to cytotoxic chemotherapy, and platinum-based doublets remain the preferred systemic therapy in advanced settings. Gemcitabine- or paclitaxel-based regimens combined with platinum agents are commonly recommended (12). In contrast to conventional NSCLC, canonical oncogenic driver alterations such as EGFR, ALK, and ROS1 are rarely detected in PPLELC, limiting the applicability of targeted therapies and further supporting the exploration of immunotherapeutic strategies.

Although several small retrospective studies have reported encouraging responses to immune checkpoint inhibitors (ICIs) in advanced PPLELC, including occasional complete pathological responses (4), current evidence remains limited to small cohorts. Robust prospective validation is lacking, particularly in the perioperative context. The optimal integration of systemic therapy with definitive local treatment for stage III disease has yet to be defined. Traditional neoadjuvant chemotherapy alone yields modest benefit, and data regarding neoadjuvant immunotherapy in this rare subtype are extremely scarce.

Ivonescimab is a first-in-class bispecific antibody that simultaneously targets PD-1 and VEGF (3). Beyond its central role in angiogenesis, VEGF is a key mediator of tumor-associated immunosuppression. It impairs dendritic cell maturation, promotes recruitment of regulatory T cells (Tregs) and myeloid-derived suppressor cells (MDSCs), and suppresses cytotoxic T-cell activity. Inhibition of VEGF signaling has been shown to normalize tumor vasculature, decrease interstitial pressure, and enhance infiltration of immune effector cells, thereby improving both drug delivery and immune activation (14, 15). Consequently, concurrent blockade of PD-1 and VEGF pathways may exert synergistic antitumor effects by simultaneously reversing immunosuppression and enhancing antitumor immune responses, providing a strong mechanistic rationale for this combinatorial approach. In the present case, the resected tumor bed showed fibrosis, focal necrosis, dense inflammatory infiltration, histiocytic and multinucleated giant-cell reaction, cholesterol cleft formation, neovascularization, and tertiary lymphoid structure formation, with no residual viable tumor cells. These findings are consistent with treatment-related regression changes and may reflect an immune-mediated pathological response after neoadjuvant chemoimmunotherapy. Similar immune-related regression patterns, including inflammatory infiltration, macrophage/histiocytic reaction, tertiary lymphoid structures, tissue-repair changes, and fibrosis, have been described in resected NSCLC specimens after neoadjuvant PD-1 blockade. In this EBV-positive, PD-L1-high tumor, these pathological changes provide a plausible correlate for the observed pCR after dual PD-1/VEGF blockade.

In stage III NSCLC, neoadjuvant chemotherapy alone achieves a pathological complete response (pCR) rate of approximately 5%, whereas neoadjuvant chemoimmunotherapy has increased pCR rates to over 20–30% (16). In the present case, neoadjuvant therapy consisting of a PD-1/VEGF bispecific antibody combined with platinum-based chemotherapy resulted in pCR in a patient with stage IIIC PPLELC. This outcome is particularly noteworthy given the historically limited efficacy of chemotherapy alone and may reflect the intrinsic immunogenicity of EBV-associated tumors in conjunction with high PD-L1 expression.

Given the high PD-L1 TPS of 70%, immunotherapy-alone or chemotherapy-de-escalated strategies may be biologically plausible. However, evidence supporting neoadjuvant immunotherapy alone in PPLELC is currently limited to isolated reports or extrapolation from NSCLC, and no prospective perioperative data are available for this rare subtype. In this patient, the initial presentation was stage IIIC disease with suspected N3 nodal involvement and a low likelihood of upfront R0 resection. Therefore, chemoimmunotherapy was selected to maximize cytoreduction, increase the probability of complete resection, and address potential micrometastatic disease. This rationale is also supported by perioperative NSCLC trials showing substantially higher pCR rates with chemoimmunotherapy than with chemotherapy alone. In CheckMate 816, neoadjuvant nivolumab plus chemotherapy achieved a pCR rate of 24.0%, compared with 2.2% with chemotherapy alonev (17). In the AEGEAN trial, perioperative durvalumab plus chemotherapy improved pCR to 17.2% versus 4.3% with chemotherapy alone (18).

Several limitations should be acknowledged. First, this is a single case report with a relatively short follow-up period, and the long-term durability of response remains unknown. Second, pretreatment PET-CT, EBUS-TBNA, and supraclavicular lymph node biopsy were not performed; therefore, nodal staging was based on radiological and ultrasonographic assessment rather than pathological confirmation. Third, repeat invasive nodal staging was not performed after neoadjuvant therapy, and surgical decision-making was based on PET-CT findings and multidisciplinary assessment. These limitations should be considered when interpreting the clinical significance of this case.

To our knowledge, this report represents the first documented case demonstrating that neoadjuvant therapy with a PD-1/VEGF bispecific antibody (ivonescimab) combined with chemotherapy may be safe, feasible, and may induce a deep pathological response selected patients locally advanced, EBV-positive, PD-L1–high PPLELC. Achievement of pCR enabled successful radical resection and highlights the potential of biomarker-informed, mechanism-driven multimodal therapy in rare thoracic malignancies. The present case should be interpreted as a preliminary single-case observation. Although pCR and R0 resection were achieved after neoadjuvant chemoimmunotherapy, the follow-up duration remains limited. Therefore, no definitive conclusions can be drawn regarding recurrence-free survival, overall survival, or the durability of response. This experience supports further investigation of dual immune–angiogenic blockade in this setting and underscores the importance of molecular-pathological stratification and multidisciplinary collaboration in optimizing outcomes for patients with PPLELC.

## Data Availability

Publicly available datasets were analyzed in this study. This data can be found here: The original contributions presented in the study are included in the article/supplementary material. Further inquiries can be directed to the corresponding authors.
